# Urban tropical forest islets as hotspots of ants in general and invasive ants in particular

**DOI:** 10.1038/s41598-022-16243-x

**Published:** 2022-07-14

**Authors:** T. P. Rajesh, K. Manoj, U. Prashanth Ballullaya, V. K. Shibil, G. Asha, Sangeetha Varma, Prabitha Mohan, Palatty Allesh Sinu

**Affiliations:** Central University of Kerala, Periya, Kerala 671316 India

**Keywords:** Ecology, Urban ecology

## Abstract

Urbanization is a crucial driver of environmental and biodiversity change. It is suggested that urbanization favours generalist and invasive species and might harm specialists of natural and semi-natural habitats. In this study, we examined how an urbanization gradient and environmental gradients in the habitat area, habitat diversity, elevation, and proportion of built-up area influenced the abundance and richness of ants within tropical forest islet habitat in south India. We used abundance (proportional trap incidence) of overall ants, native ants, invasive ants, and *Anoplolepis gracilipes*—a globally notorious invasive ant of possible south Asian origin—and rarefied richness as the response variables. We found that native ant abundance was greater and *A. gracilipes* abundance was lesser in less-urbanized landscape compared to moderately-urbanized and highly-urbanized landscape. The richness of ants and abundance of overall and invasive ants were unaffected by the urbanization. We also found that none of the measured environmental gradients but habitat diversity influenced abundance of overall ants, native ants, overall invasive ants, and richness of ants; however, *A. gracilipes* abundance was negatively correlated with habitat diversity. Ant species composition of less-urbanized landscape was distinct from that of higher urbanization levels. The richness and abundance of native ants and abundance of non-*A. gracilipes* invasive ants decreased with the abundance of *A. gracilipes*. Because the forest islets of all three urbanization levels supported similar richness of native ants, the urbanization seems not to have an adverse effect for the native ants of native forest islets. The increasing population of *A. gracilipes* in urban green islets, however, is a concern. Future studies might investigate its effect on other invertebrates of epigeal and soil strata.

## Introduction

Insects are a crucial component of terrestrial biodiversity and the facilitators of major ecosystem services and functions of tropical forests, such as nutrient cycling and soil mineralization, biological control of insect pests, and pollination among others^1^. Nevertheless, recent studies alert that insects face considerable threats from forest fragmentation, land-use change and agricultural intensification, and other global changes, such as global warming, climate change, and urbanization^2–4^. Insects are declining by their abundance, species richness, diversity, and biomass and are thinning on their populations in various ecological communities^2–4^.

Anthropogenic land-use change is a significant driver of insect decline in tropical and temperate parts and developing and developed parts of the world^3^. Agricultural intensification was a significant driver of land-use change until recently^5^. However, urbanization has taken over as a potential driver of land-use and biodiversity change in the present times^6,7^. Monitoring the patterns of biodiversity in urban ecosystems, therefore, can inform how species might respond to future global changes.

Insects, and ants in particular, are the crucial components of terrestrial biodiversity. They are dominant by their numbers and diverse by their species and functional roles in ecosystems^8–15^, which make them one of the crucial ecosystem engineers of natural, semi-natural, agricultural, and urban ecosystems^10,11^ and a suitable taxon to examine the effects of land-use change, forest fragmentation, degradation of habitats, global warming, climate change, and urbanization^16–23^. Nevertheless, ants also include some of the globally notorious invasive species^15^, which allows one to examine the effect of invasive ants on the native ants and other elements of terrestrial invertebrate diversity.

Urbanization has been shown to be associated with a change in composition and structure of native insects, dominance of certain generalist and invasive species, and invasion of invasive and alien species^24^. For ants, urbanization has been associated with a decrease^16–18^, no change^25^, or an increase^21–23,26^ of species richness and abundance. In cities, natural forest fragments, semi-natural gardens, homeyards, well-managed botanical gardens and parks that use no hazardous chemicals to manage insect pests or rodents are particularly rich and abundant on native ant species^21–23,26^. Sacred groves—the relics of biodiversity-rich natural forests^27–29^ have a higher ant diversity^25,30,31^. Although a good number of studies are in agreement that urbanization can change the characteristics of ant communities^16–22^ and favour invasive ant species in urban green spaces^15,30,32,33^, Nooten et al.^26^ found that the sacred groves of urban Hong Kong encompass a higher richness of native and specialist ants, which are resilient to invasive species. Invasion of tramp or invasive species results in colonization and spill-over to neighbouring less-disturbed areas, where they might displace or affect the activities of native ants^15,30,32,33^. Our pilot studies showed that the plausible native, but globally notorious invasive ant species, the Yellow-Crazy Ant (*Anoplolepis gracilipes*), increased several folds in urban sacred groves and spilled over to the rural sacred groves of northern Malabar region over a period of 6 years^30,34^. Conversely, in the southern United States, the invasive fire ant—*Solenopsis invicta*—translocated the native fire ant—*S. geminata*—from the natural forests to urban green spaces^33^. Therefore, the effect of urbanization on ant activities could be site, species, and community specific.

In the present investigation, we examined how an urbanization gradient and environmental gradients in the habitat area, habitat diversity, elevation, and proportion of built-up area influenced the abundance and richness of epigeic ant communities of sacred groves—natural tropical evergreen forests protected by the religious sentiments—in south India. We specifically asked the following questions: (1) what is the effect of urbanization and other habitat gradients on abundance, richness, and community structure of overall epigeic ants? (2) whether the urbanization favours the invasion of invasive ants in general and Yellow-Crazy Ant in particular? and (3) whether the native ant communities of the sacred groves were resilient to the invasion by the Yellow-Crazy Ants? In the entire study, we gave a special status for the Yellow-Crazy Ant because it is one of the 100 globally notorious invasive species^35^ and a rapidly range expanding plausible native species of south India^30,31,34^.

## Methods

### Study sites

**Figure 1:**
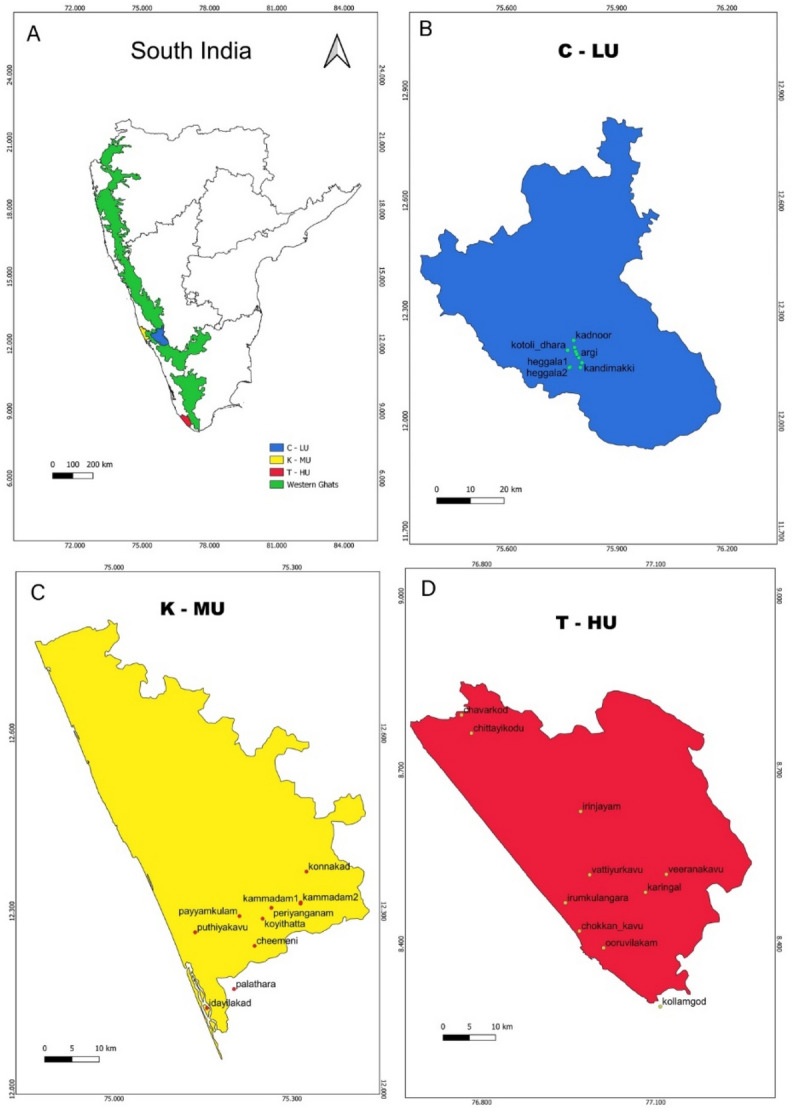
Map of the study locations. (**A**) Peninsular India shows the mountain chains of the Western Ghats biodiversity hotspot and the three locations; (**B**) the map of Kodagu district (less-urbanized location—C-LU) shows the sampling sites; (**C**) the map of Kasaragod district (moderately-urbanized location—K-MU) shows the sampling sites; (**D**) the map of Trivandrum district (highly-urbanized location—T-HU) shows the sampling sites. Map is created in QGIS version 2.8.3.

To test for the association between urbanization and ant activities of the sacred groves, we selected thirty sacred groves across three districts—Trivandrum, Kasaragod, and Kodagu—in the southern Western Ghats biodiversity hotspot that experience the urbanization of different degrees (Fig. 1). The minimum and maximum aerial distance between two locations were 70 km (Kodagu and Kasaragod) and 450 km (Kasaragod and Trivandrum). Kodagu is located on the forested landscape of the Western Ghats at an elevation of > 700 m asl. Kasaragod and Trivandrum are located on the fringes of the Western Ghats at an average elevation between 19 m asl and 135 m asl (Table S1). In each of these locations, ten independent sacred groves (hereafter, sites) were selected. The mean distance between two independent sites was about 8 km for Kodagu, 14 km for Kasaragod and 13 km for Trivandrum.

### Sacred groves

Historically, the sacred groves of Kerala and Karnataka in south India are the remnants of the natural forests that have been instituted by the religious sentiments during the widespread deforestation practices happened during the colonial period centuries ago. Therefore, their age and the way they are worshipped and conserved are the same. However, due to post-independent agricultural intensification, human migrations, and urbanizations, the sacred groves have been isolated as green islets in the middle of anthropogenically altered landscapes. Regardless of the locations, the sacred groves contain relics of evergreen forests of the size < 10 ha. While the floristic aspects of the biodiversity of the sacred groves of these States are well-known^36,37^, with our investigations, the faunistic aspects of the biodiversity of the sacred groves are just coming to the light^30,31,34,38–40^. While the sacred groves of Kodagu are located on a land mosaic of human settlements, business districts, homeyards, coffee plantations, and protected forests, the sacred groves of Kasaragod and Trivandrum are located on a land matrix of human settlements, business districts, and Coconut-based orchards or homeyards.

### Urbanization

On the degree of urbanization, Trivandrum (T-HU) represents the highly-urbanized district with a population of 1509 people per km^2^, Kasaragod (K-MU) represents the moderately-urbanized district with a population of 656 people per km^2^, and Kodagu (C-LU) represents the less-urbanized district with a population of 135 people per km^2^. Urbanization, however, was quantitively assessed by calculating the proportion of the total area under built-up conditions in one-kilometre radius from the boundary of the sacred groves into the surrounding landscape. We used remote sensing techniques to calculate the proportion of the built-up area. We used data from the Sentinel 2 procured from USGS Earth Explorer (https://earthexplorer.usgs.gov) for the present study. Images of SENTINEL 2A were used for Kodagu and Kasaragod and images of SENTINEL 2B were used for Trivandrum. Supervised classification using Maximum Likelihood Classifier was conducted to estimate the built-up area, which was measured on a hectare scale. Normalized Difference Vegetation Index (NDVI) was calculated for all the sacred groves, which indicates the vegetation health/ habitat diversity of the sacred groves. NDVI = (NIR − RED)/(NIR + RED), and the values ranged from − 1.0 to 1.0.

### Ant sampling

Ants were sampled during the dry (February–March) and wet seasons (August–September) of 2017. Together, we installed a total of 600 pitfall traps (plastic jars of 10 cm diameter and 15 cm depth) in the thirty sacred groves, but retrieved only 588 traps as some were damaged or lost to the public/wildlife. In each sacred grove, ten pitfall traps were installed on a linear transects in the core area of the groves. We installed the traps in a zigzag fashion along a linear transect. The inter-trap distance was about fifteen meters. We negotiated fallen trees, rainwater channels, termite mounds, ant nests, or any other disturbances that can affect the trap catches, while selecting spots for installing the traps. The traps were installed flush with the ground in such a way that the trap mouth opens at the ground level. We used 50 ml of 70% isopropanol as a preservative in the pitfall traps. The traps were operated for 5 days continuously, retrieved on the sixth day. We poured the contents in a glass Petri dish, and sorted the ants out from the rest of the catches and debris using a 40× stereo zoom microscope.

The ants were either identified to species or to genus and morphospecies for complex groups such as *Pheidole* spp. following Bingham^41^ and other species descriptions available in literature^42^. Ant species were subsequently grouped into native ants, invasive ants other than the Yellow-Crazy Ant (hereafter, non-YCA invasive ants), and the Yellow-Crazy Ant.

### Environmental variables

To determine the important local environmental variables and the possible ecological correlates of ant communities in the sacred groves, we collected data on forest area (from the records of the authorities), canopy closure level (on ranked visual scales^43^), number of big trees (> 30 cm DBH) and all trees of the entire sacred groves, and leaf-litter bed size for each of the thirty sacred groves during the dry season of sampling. To measure the canopy closure level and leaf litter bed depth, four spots in the vicinity of the linear transect were used. The canopy closure level was visually estimated on a percentage scale and the litter bed depth was measured using a steel centimeter ruler. The mean values of the four quadrants were used in the statistical models. In order to determine the best ecological covariates of ant activities in the models, we examined the relationships among these variables using the generalized/linear models with appropriate distribution type fitted as the error in the models. The litter depth (F_1,28_ = 4.45, p = 0.043; R^2^ = 0.11), canopy closure level (F_1,28_ = 11.35, p = 0.002; R^2^ = 0.26), number of all trees (F_1,28_ = 115, p < 0.00005; R^2^ = 0.79), and number of big trees in the sacred groves (F_1,28_ = 70.5, p < 0.00005; R^2^ = 0.71) increased with the area of the forest. Therefore, in the predictive models we used the forest area as a local driver of the ant activities in the sacred groves.

### Statistical analyses

Generalized/ Linear Models^44^ were used to examine whether the local and landscape environmental variables associated to sacred groves varied for the locations of three urbanization levels. Wherever the count was the nature of response variable, Poisson distribution was fitted and wherever the response variable was continuous in nature, Gaussian distribution was fitted as the error. The significance of the models was tested using one-way ANOVA available in the R-package, *car*^45^. The sampling completion was examined using individual-based sample coverage estimator of species richness for each location using the R-package iNEXT^46^.

We were interested in understanding how urbanization level affected ant abundances and richness. Site was considered as an independent replicate; therefore, ant data from two seasons were combined to form a single site × species matrix. Because the number of individuals collected in pitfall traps could be affected by the proximity of the traps to nests, we used trap incidence to construct the site × species matrix^47^. To account for the loss of pitfall traps to wildlife and people in some sites, the proportional trap incidence was used in the matrix. Therefore, proportional trap incidence was used as the representative of abundance in the whole analyses. The proportional trap incidence of a species was calculated by dividing the number of traps that captured individuals by the total number of traps retrieved at each site. The proportional trap incidence of each species was bounded between 0 and 1.

To determine how ant abundance per site was affected by urbanization level, proportional trap incidence of all species was averaged and used as the metric of ant abundance at the site level. We repeated this to find out the abundance of overall ants, native ants, overall invasive ants, Yellow-Crazy Ant, and invasive ants but Yellow-Crazy Ant. To determine how the diversity of ant species was affected by urbanization level, we estimated species richness rarefied by the minimum incidence of ants at each site. The rarefied richness was highly correlated with the observed richness (R^2^_adj_ = 0.61). We were also interested in understanding how the measured landscape variables were driving the ant variables along with the urbanization level; therefore, we fitted proportion of built-up area around the sacred groves, habitat diversity of sacred groves (NDVI), area of the sacred groves, and elevation of the sites as the covariates in the models. We used a linear model (lm) with the urbanization level (highly-urbanized/ moderately-urbanized/less-urbanized), NDVI of sacred grove, elevation, area of sacred grove, and proportion of built-up area around sacred groves as the predictor variables. We arcsine square-root transformed the proportional trap incidence of each ant variable but the rarefaction estimates of species richness, square-root transformed the NDVI, and log-transformed the elevation and area of sacred groves to meet the assumptions of linear model fitting. We did not find significant interactions among the predictors; therefore the final fitted model was “model < − lm(asin(sqrt(y)) ~ urbanization level + sqrt(proportion of built-up area) + sqrt(NDVI) + log(area of sacred grove) + log(elevation))”. The significance of the overall model was tested using Anova available in the R-package, *car*^45^. The residuals of the fitted final models were tested using the R-package DHARMa^48^.

We were interested in understanding whether the abundance of Yellow-Crazy Ant in sites was driving the abundance of native ants and other invasive ant species. To represent the abundance of both the ants, proportional trap incidences were used in the linear models. The proportional trap incidence of native or other ants were used as the response variable and proportional trap incidence of Yellow-Crazy Ant was used as the predictor. Both the variables were arcsine-square-root transformed to meet the assumptions of linear modelling.

To test for the differences in the ant community composition among sites of different urbanization gradients, we used a paired permutational multivariate analysis of variance (PERMANOVA) using the ‘adonis’ function and 999 permutations. This analysis was performed in the R-package ‘vegan’^49^. In the PERMANOVA, the Jaccard distance matrix of species composition of overall ants was used as the response variable, with the urbanization level (highly-urbanized, moderately-urbanized, and less urbanized) as a fixed factor. In the model, the strata argument was set to ‘site pair’ for the randomizations to occur within each pair. We performed this analysis for overall ants. We used non-metric multidimensional scaling (NMDS) available in the R-package ‘vegan’ to visualize the variations in ant community among urbanization levels. All analyses were performed in R v.3.2.3^50^.

## Results

### Species diversity

**Figure 2:**
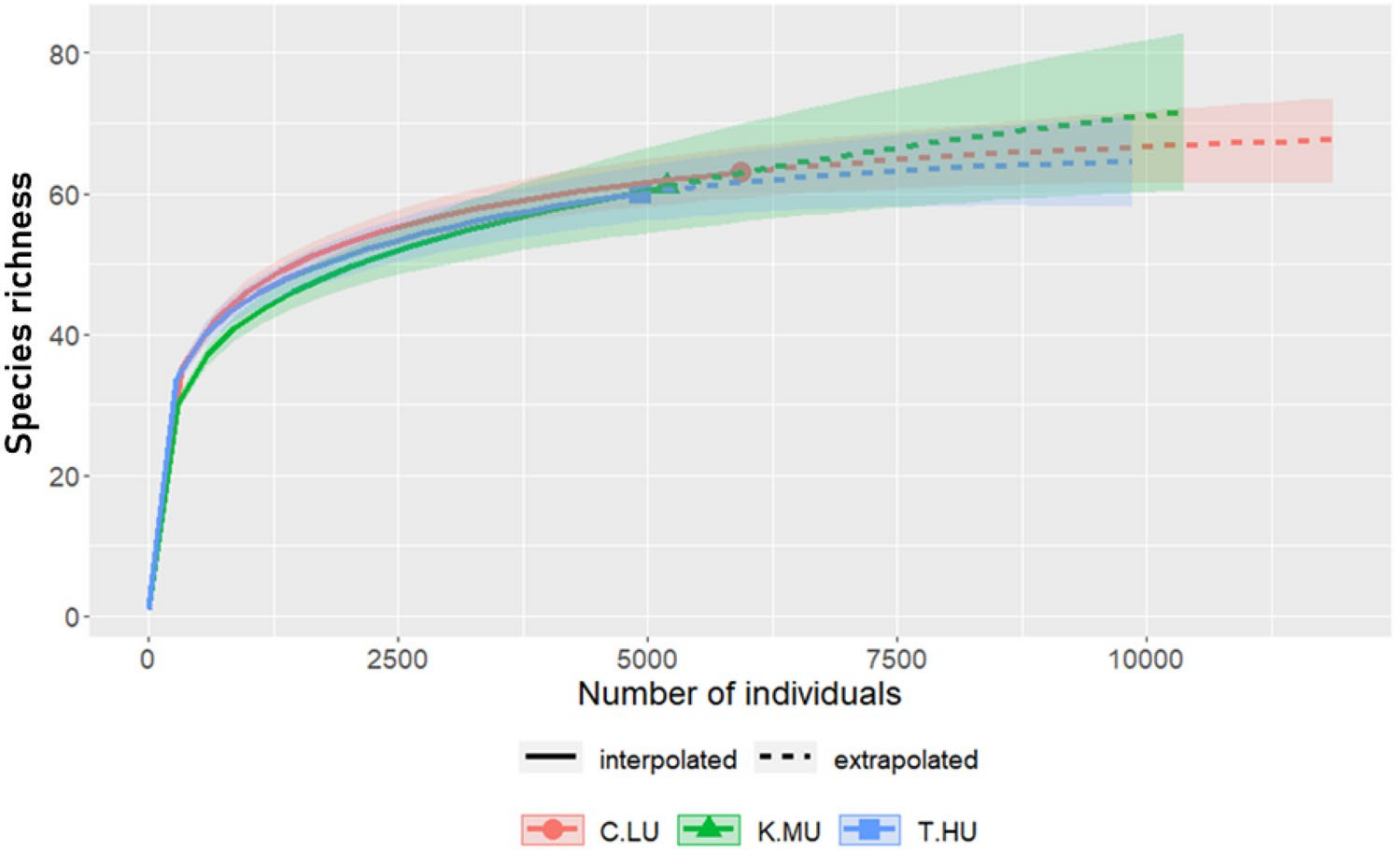
Individual-based rarefaction plots show the observed and rarefied species richness in three locations. C.LU = less-urbanized Kodagu; K.MU = moderately-urbanized Kasaragod; T.HU = highly-urbanized Trivandrum.

Sixteen thousand forty-one ants belonging to 96 species and eight subfamilies (Table 1 and Table S2) were collected. The rarefaction and sample coverage estimators suggest that the present sampling was near to completion (Fig. 2; Fig. S1). Out of overall ants collected, 11,823 individuals (74%) belonged to 86 species and 4,208 individuals (26%) belonged to 10 species were native and invasive ants, respectively. Of the ten invasive species collected in the study, the Yellow-Crazy Ant’s share alone was about 50% (Table 1). *Lepisiota opaca, Monomorium floricola, Monomorium monomorium, Odontomachus haematodus, Paratrechina longocornis, Solenopsis geminata, Tapinoma melanocephalum, Tetramorium bicarinatum,* and *Tetramorium lanuginosum* were the other invasive ant species collected in the study. Myrmicinae with 46 species, Formicinae with 20 species, Ponerinae with 17 species, and Dorylinae with eight species were the speciose and abundant ant subfamilies in the sample; Dolichoderinae, Leptanillinae, Amblyoponinae, and Ectatomminae were collected by one or two species (Table S3).

**Table 1:** Summary of ants sampled in three locations representing the levels of urbanization (LU = less-urbanized; MU = moderately-urbanized; HU = highly-urbanized).

Location	Abundance	Richness	Native ant abundance	Native ant richness	Invasive ant abundance	Invasive ant richness	*A. gracilipes* abundance
Kodagu (LU)	6.56	63	5.3	56	1.3	7	0.02
Kasaragod (MU)	5.2	61	3.6	52	1.6	9	0.42
Trivandrum (HU)	4.7	60	3.11	53	1.6	7	0.6
Overall	5.5	96	3.99	86	1.5	10	0.35

The percent share of native and invasive ants is given. The percent share of *A. gracilipes* is on the total abundance of ants.

### Environmental variables

The sacred groves of the three locations were different on the physiographical features and vegetation; the area of the sacred groves (*F*_*2,27*_ = 4.23,* P* = 0.02), the number of all trees (*F*_*2,27*_ = 6.4, *P* = 0.005), and the number of big trees in the sacred groves (*F*_*2,27*_ = 10.3,* P* < 0.005) were markedly lower in highly-urbanized locations than moderately- and less-urbanized locations (Fig. S2). However, the canopy closure level (*F*_*2,27*_ = 1.64, *P* = 0.21) and the leaf litter depth (*F*_*2,27*_ = 2.7, *P* = 0.08) of the sacred groves of the three locations were similar (Fig. S2). The habitat diversity (NDVI) of the sacred groves of the highly-urbanized location was lower than the moderately- and less- urbanized locations (*F*_*2,27*_ = 5.22,* P* = 0.01). However, the proportion of the built-up area around the sacred groves was higher in highly-urbanized locations than the locations of moderate and less urbanization (*F*_*2,27*_ = 14.39, *P* < 0.005). The NDVI of the sacred groves decreased with the increasing proportion of built-up area around the sacred groves (− 0.38 ± 0.06, *Z* = − 6.78, *P* < 0.0005; *R*^2^ = 0.61; Fig. S3).

### Ant abundance and richness along the urbanization gradient

**Figure 3:**
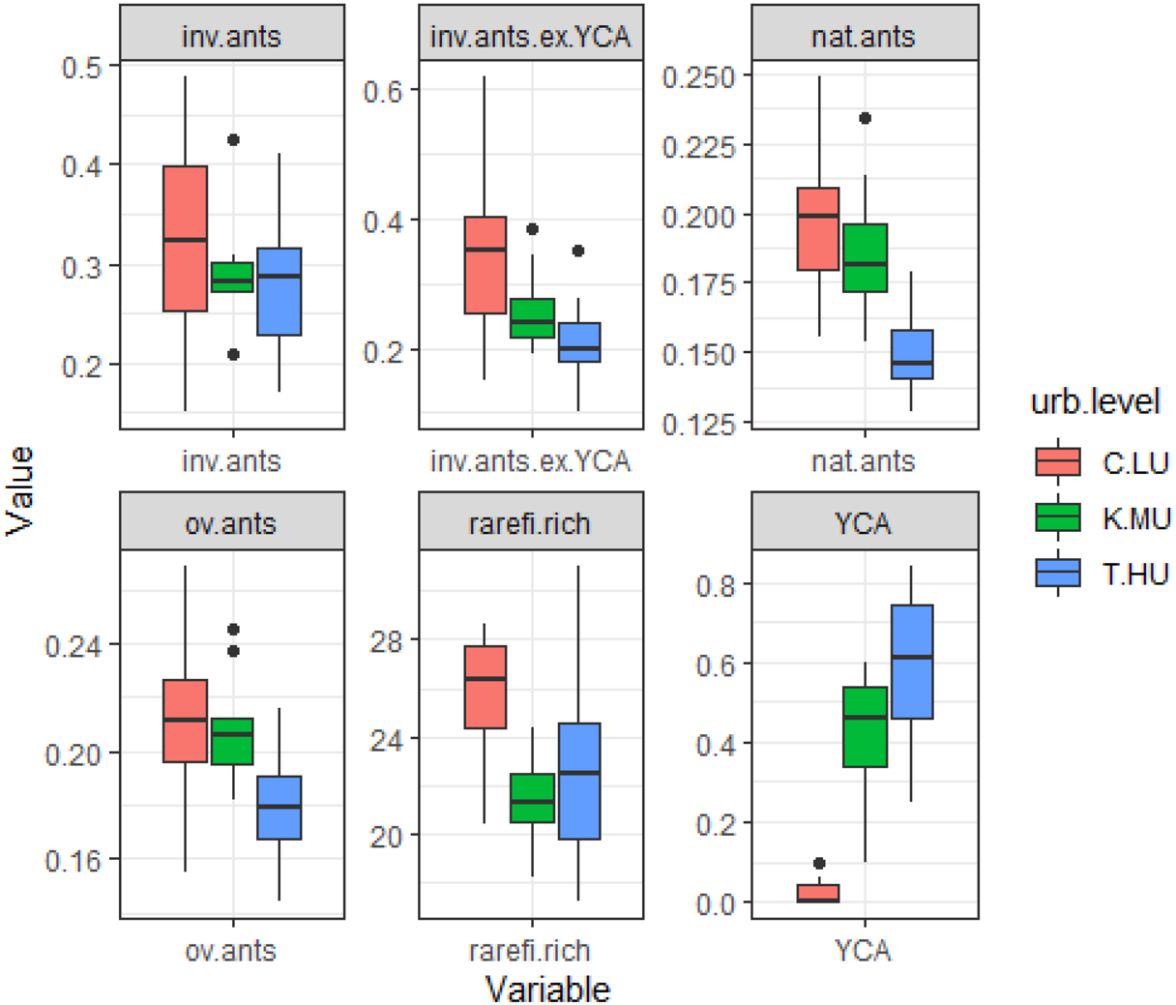
Effects of urbanization gradient on ant variables. Proportional trap incidence is used as the measure of abundance. inv.ants = overall invasive ants; inv.ants.ex.YCA = non-*A. gracilipes* invasive ants; nat.ants = native ants; ov.ants = overall ants; rarefi.rich = Richness of native ants; YCA = abundance of Yellow-Crazy Ant (*A. gracilipes*). Urbanization levels: C.LU = less-urbanized Kodagu; K.MU = moderately-urbanized Kasaragod; T-HU = Highly-urbanized Trivandrum.

The abundance of native ants (*F*_2,23_ = 5.07, *P* = 0.01) decreased with the level of urbanization (Table 2, Figs. 3 and S4). On the other hand, the abundance of *A. gracilipes* increased with the level of urbanization (*F*_2,23_ = 3.99, *P* = 0.03; Fig. 3 and Fig. S4). The sacred groves of all the three urbanization levels, however, had similar abundance of overall ants (*F*_2,23_ = 2.3, *P* = 0.12) and richness of native ant species (*F*_2,23_ = 1.7, *P* = 0.19). Similarly, the abundance of overall invasive ants and non-*A. gracilipes* invasive ants was similar in the sites of the three urbanization levels (Figs. 3 and S4). None of the measured covariables but the habitat diversity of the sacred groves (NDVI) affected the ant variables (Table 2, Fig. S2). The abundance of *A. gracilipes* decreased with the NDVI of the sacred groves (*F*_2,23_ = 4.7, *P* = 0.04).

**Table 2:** Parameter estimates from the linear models analyzing the responses of ant variables to landscape and local variables.

	Overall ant abundance		Native ant abundance		Invasive ant abundance		Non-YCA invasive ant abundance		*A. gracilipes* abundance		Rarefied richness	
	F	P	F	P	F	P	F	P	F	P	F	P
Urbanization level	2.3	0.12	5.07	0.01	0.3	0.7	0.67	0.5	12.3	0.0002	1.7	0.16
√ built-up area	0.03	0.8	0.4	0.5	0.04	0.8	0.01	0.9	0.9	0.9	0.004	0.24
Log (elevation)	0.0005	0.99	0.3	0.6	0.6	0.4	0.3	0.6	0.6	0.6	0.22	0.36
Log (SG area)	0.005	0.94	0.07	0.8	0.16	0.7	0.003	0.95	0.7	0.5	0.99	0.76
√NDVI	0.11	0.7	0.3	0.6	1.44	0.2	0.08	0.7	4.7	0.04	2.72	0.42
R^2^_adj_	0.14		0.4		− 0.06		0.12		0.82		0.21	
F (6,23)	1.8		3.7		0.77		1.6		23.5		2.3	
P	0.14		0.009		0.6		0.18		< 0.0005		0.06

### Ant communities in the sacred groves along the urbanization gradient

**Figure 4:**
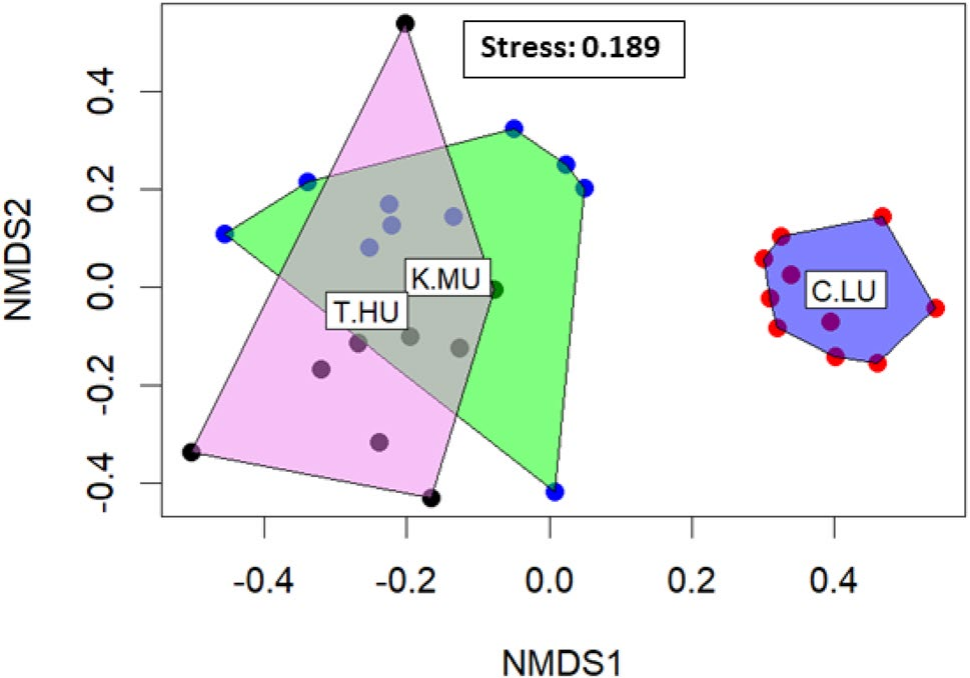
The NMDS plots illustrate the sacred groves of three urbanization levels on ant community (presence-absence data). Plots: T.HU = highly-urbanized Trivandrum; C.LU = less-urbanized Kodagu; and K.MU = moderately-urbanized Kasaragod.

The epigeic ant communities of the sacred groves of the three urbanization levels was different (PERMANOVA: Pseudo-*F* = 6.4, *R*^*2*^ = 0.32, *P* = 0.001; Fig. 4); the less-urbanized sites stood markedly away from the highly-urbanized and moderately-urbanized sites. Twenty-one species of ants belonged to Myrmicinae (11 species), Ponerinae (4 species), Formicinae (5 species), and Dolichoderinae (1 species) dominated the entire ant community of the sacred groves of the three urbanization levels. Among the ten most dominant ant species of the respective ant communities (Fig. S5), five species belonged to Myrmicinae (*Pheidole sp15, Pheidole sp18, Tetramorium mixtum*), Formicinae (*A. gracilipes*) and Ponerinae (*Odontomachus haematodus*) were common to moderately-urbanized and highly-urbanized sites, three species belonged to Myrmicinae (*Pheidole sp15*) and Formicinae (*Oecophyla smaragdina* and *Nylanderia indica*) were common to less-urbanized- and moderately-urbanized sites, and one species belonged to Myrmicinae (*Pheidole sp15*) was common to less urbanized and highly urbanized sites. The latter species was also common in the sacred groves of all the three urbanization levels and was the most collected one in the highly-urbanized and moderately-urbanized sacred groves. However, in the highly-urbanized sacred groves, *A. gracilipes* replaced *Pheidole sp*15 by a remarkably higher margin (Fig. S5).

### Effect of Yellow-Crazy Ant on other invasive ants and native ants

In all the three locations, one or several species of invasive ants were present in the sacred groves (Figs. S5 and S6). As far as *A. gracilipes* (Yellow-Crazy Ant) was concerned, it was collected only in three out of the ten sacred groves of the less-urbanized landscape but was collected in all the ten sacred groves of the moderately- and highly-urbanized landscapes. The ratio of *A. gracilipes* to the native ants on the proportional trap incidence of ants increased from the less-urbanized sacred groves (0.02) to moderately-urbanized (0.44) and highly-urbanized sacred groves (0.61). The share of *A. gracilipes* in the abundance of overall invasive ant species was 2.7%, 55%, and 86% in less-, moderately-, and highly-urbanized locations, respectively. The proportional trap incidence of *A. gracilipes* and *Paratrechina longocornis* (Longhorn-Crazy Ant) decreased from the highly-urbanized sacred groves to the less-urbanized sacred groves. For *Monomorium floricola* and *Tetramorium lanuginosum*—the other two invasive species—the proportional trap incidence increased from the highly-urbanized sacred groves to the less-urbanized sacred groves. *Tapinoma melanocephalum* was collected similarly in the sacred groves of all three urbanization levels. *Odontomachus haematodus* was not collected in the sacred groves of less-urbanized sacred groves, but collected most in the moderately-urbanized sacred groves (Fig. S6).

The proportional trap incidence of native ants decreased with the proportional trap incidence of *A. gracilipes* (*F*_*1,28*_ = 6.07, *P* = 0.02; *R*^*2*^*adj* = 0.15). The richness of native ants also decreased with the abundance of *A. gracilipes* (*F*_*1,28*_ = 6.74, *P* = 0.01; *R*^*2*^*adj* = 0.17). The abundance of combined non-*A. gracilipes* invasive ants decreased with the abundance of *A. gracilipes* (*F*_*1,28*_ = 4.71, *P* = 0.04; *R*^*2*^*adj* = 0.17). When we analysed the relationship between *A. gracilipes* and five most dominant invasive ant species, we found that *P. longicornis* (0.25 ± 0.11, *T* = 2.3, *P* = 0.03; *R*^*2*^*adj* = 0.13) and *O. haematodus* increased (0.77 ± 0.13, *T* = 5.9, *P* < 0.005; *R*^*2*^*adj* = 0.54), *T. lanuginosum* (− 0.44 ± 0.09, *T* = 4.43, *P* = 0.0001; *R*^*2*^*adj* = 0.39) and *M. floricola* (− 0.45 ± 0.09, *T* = − 4.8, *P* = 0.00005; *R*^*2*^*adj* = 0.45) decreased and *T. melanocephalum* unchanged with the abundance of *A. gracilipes* (− 0.16 ± 0.14, *T* = − 1.13, *P* = 0.27; *R*^*2*^*adj* = 0.01; Fig. S7). The interaction between the abundance of Yellow-Crazy Ant and the proportion of built-up area was a poor driver of native ant abundance (P = 0.4) and richness (P = 0.5).

## Discussion

We tested whether the abundance and the richness of native and invasive epigeic ants of the tropical forest islets of the sacred groves were affected by the urbanization and whether the abundance of the native ants and other invasive ants was affected by the abundance of Yellow-Crazy Ant—an invasive species responded positively to the urbanization. The most striking findings of this study are that (a) the urbanization unaffected the richness, but negatively affected the abundance of native ants; (b) the urbanization, despite unaffected the abundance of overall invasive ants, favoured the abundance of *A. gracilipes*—the Yellow-Crazy Ant—and *Paratrehina longicornis*—the Longhorn-Crazy Ant—the two invasive ant species of economic and ecological importance; (c) the abundance and the richness of native ants decreased with the abundance of *A. gracilipes*; (d) the abundance of *A. gracilipes* had mixed effects for other dominant invasive ant species; and (e) the sacred groves of less-urbanized sites are different from the sacred groves of the moderately- and highly-urbanized sites on the composition of ants.

In our predictive models, we used the proportion of the built-up area around the sacred groves as a quantifiable measure of urbanization and disturbance, the NDVI as the measure of the habitat diversity of the sacred groves, the altitude and the area of the sacred groves as the potential covariates of ant abundance and diversity. We fitted altitude and area of the habitat as the drivers because they have ranged considerably in the present study and are often depicted as the drivers of ant abundance and diversity. The models, however, found that none of the co-variables were crucial predictors of the abundance and richness of overall ants and different functional guilds of the ants. However, the vegetation health of the sacred groves (NDVI) was a crucial driver of *A. gracilipes*, which was lesser for the sacred groves of higher NDVI values.

As expected, the abundance of the Yellow-Crazy Ant increased in the sacred groves with the proportion of the built-up area around the sacred groves. The trap incidence of the Yellow-Crazy Ant was highest for the sacred groves of the highly-urbanized sites. Its trap incidence in the sacred groves of the moderately-urbanized site was increasing dramatically over the years^30^. It is suggested that the invasive ants, including the Yellow-Crazy Ant increase their population quickly in more disturbed environments than the less disturbed areas in the naturalized and invaded regions^17,30,31,51^. The sacred groves of the moderately- and less-urbanized sites were similar on the vegetation health. Our findings that the Yellow-Crazy Ant was hardly collected in the sacred groves of the less-urbanized sites agree to the patterns observed for the invasive ants along the disturbance gradients by previous studies elsewhere^17,30,31,51^.

Studies often suggest that urbanization may not have an adverse effect for social insects, such as ants^22,52–54^ and bees^55^. On the diversity of the native ants of the sacred groves, urbanization seems not to have an adverse effect in the present study. The effect the urbanization had on the abundance of the native ants was marginal. These patterns suggest that the sacred groves, regardless of their size, can conserve the native ants even in the highly urbanized areas. Our findings agree with Melliger et al.^51^, who found that the size of the urban green spaces is a poor predictor of surface-active ants.

The other possible, rather an indirect, channel the urbanization can adversely affect the native ants is through favouring the population of certain invasive and tramp ant species and giving a competitive displacement of native ants^56–60^. In our study, urbanization has driven the population of *A. gracilipes*; it was collected abundantly in the highly and moderately urbanized sacred groves than the less-urbanized landscape. The urban environment is always dynamic, therefore native ants with sophisticated and modular communication skills are better suited to lead an urban life^52,61^. However, their ability to thrive in the urban green islets might be partly driven by the nature of the dominant urban-adapted native and invasive species. The findings of the present study also suggest that apart from the Yellow-Crazy Ant, the Longhorn Crazy Ant and *Odontomachus haematodus* also might have adapted to live in the urbanized natural areas. They were collected abundantly in the sacred groves of the highly urbanized landscape, but were collected by a negligible number in the less-urbanized sacred groves and moderately in the moderately-urbanized sacred groves. Although *A. gracilipes* was confined only in the three sacred groves and collected in a very few numbers of the traps in the less-urbanized landscape of Kodagu, the invasion may be observed seriously. Our 6-year-long monitoring of the Yellow-Crazy Ant in the groves and agroecosystems of Kasaragod—the moderately urbanized site of the present study—found an exponential growth and spill-over of its population to newer sites including agroecosystems and hindrance of pollination services in vegetable crops^30,31,62^.

Among the invasive ant species collected in the present study, both the species of Crazy Ants—*A. gracilipes* and *P. longicornis*—are behaviourally-sophisticated and aggressive to occupy space and monopolize resources first^63–65^. The Yellow-Crazy Ant is one of the 100 notorious invasive species and one of the world's six harmful and widespread invasive ant species. The Longhorn Crazy Ant is also one of the fast-spreading species of tropical and subtropical natural and disturbed areas^65^. They share their position with the other globally notorious invasive ants—*Solenopsis invicta* (Red Imported Fire Ant), *S. geminata* (Tropical Fire Ant), *Linepithema humile* (Argentine Ant), *Wasmannia auropunctata* (Little Fire Ant), and *Pheidole megacephala* (African Big-Headed Ant)^15,64,65^. The Yellow-Crazy Ant seems to be the native of the Asia–Pacific and Indian Ocean region. Therefore, peninsular India, where this study took place, may be one of the native ranges of *A. gracilipes*. Yet, our study suggests that the Yellow-Crazy Ant can negatively affect the abundance, richness, and community of the native ants and abundance of certain species of invasive ants. Among the dominant invasive ant species, *T. lanuginosum* and *M. floricola* decreased with the abundance of *A. gracilipes*.

Some native and invasive ant species belonged to *Pheidole*, *Tetramorium*, *Paratrechina*, *Tapinoma*, and *Monomorium* collected in the present study are known to make super-colonies and adapt quickly to the disturbed environment, such as an urban environment. While that is so, the study also collected several tiny and sluggish subterranean ants known to live in the less-disturbed environments from the sacred groves of high urbanization. For instance, an examination of *Strumigenys* species collected in the present study (three species and 191 individuals) shows that their average proportional trap incidence was higher in the sacred groves of highly-urbanized sites than the moderately- and less-urbanized sites. These species are rare and endemic to the Western Ghats biodiversity hotspot. Their collection from the sacred groves of high urbanization sites reiterates that the native forest fragments of highly urbanized sites also support rare ants of the Western Ghats biodiversity hotspots and urbanization may not have a negative effect on the native ants.

In brief, our findings suggest that the sacred groves should be the hotspots of native ants. Although the disturbances due to urbanization and invasion of the invasive species can be an imminent threat for the native ant biodiversity, the healthy sacred groves are immune to such pressures. Although species diversity is unaffected by urbanization, the present study did not assess the effect of urbanization on the functional diversity of the ants. Throwing some lights on this will inform how urbanization might affect the forest functions. Although a plausible native of South Asia, the increase in the population of the Yellow-Crazy Ant, in the lights of its direct positive effects on the population of two other invasive ant species and direct negative effects on the abundance and diversity of the native ants, may be treated seriously. Future studies should also explore how the Yellow-Crazy Ant might affect the other elements of invertebrate biodiversity, such as beetles, collembolans, and epigeal flies.

Conserving the natural forests of any size should be our priority rather than replacing them with a manipulated artificial “forest”. The sacred groves are the natural green islets, which support biodiversity, the cultural diversity, and the history of peninsular India, particularly the Western Ghats^28,29,31,39,40^. Urbanization is a significant driver of biodiversity and environmental change across latitudes and longitudes, and cities and city lifestyles are marauding the world. The cultural forests, such as sacred groves and church forests, because are conserved by the myths, beliefs, taboos, and religious reasons, can be the effective and sustainable global models of local biodiversity conservation. They would undoubtedly serve as the refuges for the urban wildlife and may be protected rather than replaced by the manipulated urban green islets or parks.

## Supplementary Information

Supplementary Information.
