# Supplementary material for: Urban tropical forest islets as hotspots of ants in general and invasive ants in particular
Source: Sci Rep. 2022 Jul 14;12:12003. doi: 10.1038/s41598-022-16243-x (PMC9283449; doi:10.1038/s41598-022-16243-x)
Supplement: Supplementary file 1 — Supplementary Information. [file 41598_2022_16243_MOESM1_ESM.docx]

**Electronic supplementary Files**

**Urban tropical forest islets as hotspots of ants in general and invasive ants in particular**

**Table S1. Site details of three locations and urbanization levels. Coorg represents the less-urbanized location; Kasaragod represents the moderately-urbanized location; and Trivandrum represents the highly-urbanized location**

| **Location** | **Site** | **Elevation** | **Latitude** | **Longitude** |
| --- | --- | --- | --- | --- |
| coorg | argi | 903 | 12.172623 | 75.79697 |
| coorg | bettoli | 902 | 12.189267 | 75.78845 |
| coorg | bettoli_ayya | 906 | 12.18263 | 75.791208 |
| coorg | heggala1 | 950 | 12.147603 | 75.773113 |
| coorg | heggala2 | 1050 | 12.14607 | 75.770934 |
| coorg | kadnoor | 880 | 12.219888 | 75.782748 |
| coorg | kandimakki | 918 | 12.159041 | 75.805326 |
| coorg | kotoli_ayyap | 900 | 12.200675 | 75.785165 |
| coorg | kotoli_dhara | 907 | 12.193038 | 75.766375 |
| coorg | perumbadi | 820 | 12.146356 | 75.800903 |
| kasaragod | cheemeni | 100 | 12.241533 | 75.236769 |
| kasaragod | idayilakad | 8 | 12.136667 | 75.1564 |
| kasaragod | kammadam1 | 106 | 12.312867 | 75.3143 |
| kasaragod | kammadam2 | 98 | 12.314917 | 75.3146 |
| kasaragod | konnakad | 102 | 12.366833 | 75.3243 |
| kasaragod | koyithatta | 120 | 12.287417 | 75.2503 |
| kasaragod | palathara | 12 | 12.168711 | 75.2021 |
| kasaragod | payyamkulam | 90 | 12.291733 | 75.2113 |
| kasaragod | periyanganam | 139 | 12.305617 | 75.2652 |
| kasaragod | puthiyakavu | 18 | 12.264483 | 75.1366 |
| trivandrum | chavarkod | 40 | 8.794819 | 76.765098 |
| trivandrum | chittayikodu | 46 | 8.763445 | 76.78206 |
| trivandrum | chokkan_kavu | 52 | 8.422162 | 76.968465 |
| trivandrum | irinjayam | 142 | 8.628476 | 76.970497 |
| trivandrum | irumkulangara | 19 | 8.470875 | 76.944219 |
| trivandrum | karingal | 90 | 8.48901 | 77.082417 |
| trivandrum | kollamgod | 17 | 8.292016 | 77.107937 |
| trivandrum | ooruvilakam | 36 | 8.393235 | 77.010192 |
| trivandrum | vattiyurkavu | 50 | 8.519201 | 76.98615 |
| trivandrum | veeranakavu | 69 | 8.520052 | 77.118432 |

**Table S2. The average proportional trap incidence of ant species collected in the sacred groves of three locations representing different levels of urbanization (C.LU=Less-Urbanized Coorg; K.MU=Moderately-Urbanized Kasaragod; T.HU=Highly-Urbanized Trivandrum)**

| Nativity | Species | C.LU | K.MU | T.HU |
| --- | --- | --- | --- | --- |
| Native | *Acropyga.acutiventris* | 0 | 0 | 0.011111 |
|  | *Aenictus.aitkenii* | 0 | 0.030263 | 0.015263 |
|  | *Aenictus.ceylonicus* | 0.005 | 0 | 0.01 |
|  | *Aenictus.wilsoni* | 0.01 | 0 | 0 |
|  | *Aenictus.wroughtonii* | 0.005263 | 0 | 0 |
|  | *Anochetus.myops* | 0.021765 | 0.020526 | 0.005263 |
|  | *Aphaenogaster.beccarii* | 0.075 | 0 | 0 |
|  | *Bothroponera.sulcata* | 0.066579 | 0.081579 | 0.031608 |
|  | *Brachyponera.luteipes* | 0.13582 | 0.025 | 0.060263 |
|  | *Camponotus.angusticollis* | 0 | 0.01 | 0 |
|  | *Camponotus.compressus* | 0 | 0.005 | 0 |
|  | *Camponotus.confuci* | 0.010263 | 0.005 | 0.005263 |
|  | *Camponotus.minor1* | 0.040882 | 0.04 | 0.092368 |
|  | *Camponotus.minor2* | 0.025263 | 0 | 0 |
|  | *Camponotus.minor3* | 0.005263 | 0 | 0 |
|  | *Camponotus.variegatus.infuscus* | 0.529737 | 0.080263 | 0.031345 |
|  | *Carebara.affinis* | 0.055263 | 0.01 | 0.068304 |
|  | *Carebara.minor1* | 0.005882 | 0.005 | 0 |
|  | *Carebara.minor2* | 0 | 0 | 0.005263 |
|  | *Centromyrmex.feae* | 0 | 0 | 0.005263 |
|  | *Cerapachys.sp.eragatoid.queen* | 0.015 | 0.010263 | 0.005 |
|  | *Crematogaster.biroi* | 0 | 0 | 0.155556 |
|  | *Crematogaster.rogenhoferi* | 0 | 0.005 | 0 |
|  | *Crematogaster.wroughtonii* | 0.183251 | 0.145263 | 0.174006 |
|  | *Diacamma.assamense* | 0 | 0.035 | 0.041111 |
|  | *Diacamma.ceylonense* | 0 | 0.03 | 0.062485 |
|  | *Dolichoderus.taprobanae* | 0 | 0 | 0.01 |
|  | *Dorylus.orientalis* | 0.010263 | 0 | 0 |
|  | *Gnamptogenys.binghamii* | 0 | 0.005 | 0 |
|  | *Leptanilla.escheri* | 0 | 0 | 0.005556 |
|  | *Leptogenys.assamensis* | 0.051053 | 0.015 | 0.051579 |
|  | *Leptogenys.chinensis* | 0.415464 | 0.015 | 0 |
|  | *Leptogenys.dalyi* | 0.005 | 0 | 0 |
|  | *Leptogenys.dentilobis* | 0.104489 | 0.005263 | 0 |
|  | *Leptogenys.diminuta* | 0.112461 | 0 | 0.005263 |
|  | *Mesoponera.melanaria* | 0.23387 | 0.035526 | 0.041053 |
|  | *Myrmecina.urbanii* | 0 | 0.005 | 0 |
|  | *Myrmicaria.brunnea* | 0 | 0 | 0.026316 |
|  | *Myrmicinae.queen* | 0 | 0 | 0.005263 |
|  | *Nylanderia.indica* | 0.409799 | 0.260263 | 0.102105 |
|  | *Odontoponera.denticulata* | 0.321858 | 0 | 0 |
|  | *Oecophylla.smaragdina* | 0.292136 | 0.253158 | 0.071053 |
|  | *Parasyscia.indicus* | 0.015 | 0 | 0.005 |
|  | *Parasyscia.seema* | 0.040526 | 0.01 | 0 |
|  | *Pheidole.minor1* | 0 | 0.135789 | 0.15155 |
|  | *Pheidole.minor10* | 0.010263 | 0 | 0 |
|  | *Pheidole.minor11* | 0.005 | 0 | 0 |
|  | *Pheidole.minor12* | 0.091579 | 0.05 | 0 |
|  | *Pheidole.minor13* | 0.091672 | 0.005 | 0 |
|  | *Pheidole.minor14* | 0.051935 | 0 | 0 |
|  | *Pheidole.minor15* | 0.780418 | 0.628684 | 0.44386 |
|  | *Pheidole.minor16* | 0 | 0.005 | 0.005 |
|  | *Pheidole.minor17* | 0.190635 | 0.030263 | 0.031053 |
|  | *Pheidole.minor18* | 0.129923 | 0.412105 | 0.461696 |
|  | *Pheidole.minor2* | 0 | 0.055 | 0.045263 |
|  | *Pheidole.minor3* | 0 | 0 | 0.132924 |
|  | *Pheidole.minor4* | 0 | 0 | 0.015556 |
|  | *Pheidole.minor5* | 0.035263 | 0.26 | 0.087105 |
|  | *Pheidole.minor6* | 0.030263 | 0.005 | 0.041374 |
|  | *Pheidole.minor7* | 0.005 | 0.005 | 0 |
|  | *Pheidole.minor8* | 0.015 | 0.01 | 0.056345 |
|  | *Pheidole.minor9* | 0.005263 | 0 | 0.005556 |
|  | *Pheidole.sagei* | 0 | 0.005 | 0 |
|  | *Pheidole.spathifera* | 0.040789 | 0 | 0.010556 |
|  | *Pheidole.templaria* | 0 | 0.04 | 0 |
|  | *Pheidole.watsoni* | 0 | 0.005 | 0 |
|  | *Polyrhachis.furcata* | 0.021409 | 0 | 0 |
|  | *Polyrhachis.hippomanes.ceylonensis* | 0 | 0.005 | 0.01 |
|  | *Polyrhachis.proxima* | 0 | 0.005 | 0 |
|  | *Polyrhachis.tibialis* | 0 | 0.005 | 0 |
|  | *Ponera.indica* | 0.01 | 0 | 0 |
|  | *Ponerinae.sp* | 0.02 | 0.1 | 0.026053 |
|  | *Prenolepis.naoroji* | 0.005 | 0 | 0 |
|  | *Pseudolasius.minor* | 0.005882 | 0 | 0.005556 |
|  | *Pseudolasius.sp* | 0.005 | 0 | 0.015789 |
|  | *Pseudoneoponera.rufipes* | 0 | 0.065263 | 0.025526 |
|  | *Rhopalomastix.rothneyi* | 0.021146 | 0.116053 | 0.030263 |
|  | *Stigmatomma.minutum* | 0 | 0 | 0.005 |
|  | *Strumigenys.fixata* | 0.081672 | 0.060263 | 0.124269 |
|  | *Strumigenys.hostilis* | 0.051409 | 0.050526 | 0.051608 |
|  | *Strumigenys.lyroessa* | 0.046409 | 0.02 | 0.062456 |
|  | *strumigenys.queen* | 0 | 0 | 0.005 |
|  | *Tetramorium.keralense* | 0.112817 | 0.035263 | 0.020263 |
|  | *Tetramorium.mixtum* | 0.236687 | 0.295789 | 0.141111 |
|  | *Tetramorium.rugigaster* | 0.025882 | 0 | 0 |
|  | *Tetramorium.sentosum* | 0.015263 | 0.035526 | 0 |
| invasive | *Anoplolepis.gracilipes* | 0.021146 | 0.427368 | 0.598889 |
|  | *Lepisiota.opaca* | 0 | 0.035 | 0 |
|  | *Monomorium.floricola* | 0.514443 | 0.211316 | 0.119064 |
|  | *Monomorium.monomorium* | 0.015 | 0.005 | 0 |
|  | *Odontomachus.haematodus* | 0 | 0.537632 | 0.380409 |
|  | *Paratrechina.longicornis* | 0.041765 | 0.010263 | 0.220263 |
|  | *Solenopsis.geminata* | 0.005 | 0.01 | 0 |
|  | *Tapinoma.melanocephalum* | 0.28291 | 0.210526 | 0.143947 |
|  | *Tetramorium.bicarinatum* | 0 | 0 | 0.020789 |
|  | *Tetramorium.lanuginosum* | 0.43918 | 0.165 | 0.087398 |
|  | Observed abundance | 5931 | 5185 | 4925 |
|  | Observed richness | 63 | 61 | 60 |

**Table S3. The distribution of ants in different subfamilies. The share of subfamilies in ant abundance is given in parentheses**

| Subfamilies | Overall abundance | Overall abundance | Overall richness | Native abundance | Native richness | Invasive abundance | Invasive richness | *A.gracilipes* |
| --- | --- | --- | --- | --- | --- | --- | --- | --- |
| Amblyoponinae | 1 (0.006%) | 1 | 1 | 1 | 1 | 0 | 0 | - |
| Dolichoderinae | 268 (1.7%) | 268 | 2 | 2 | 1 | 266 | 1 | - |
| Dorylinae | 1013 (6%) | 1013 | 8 | 1013 | 8 | 0 | 8 | - |
| Ectatomminae | 1 (0.006%) | 1 | 1 | 1 | 1 | 0 | 0 | - |
| Formicinae | 3528 (22%) | 3528 | 20 | 1291 | 17 | 2237 | 3 | 2123 |
| Leptanillinae | 2 (0.01%) | 2 | 1 | 2 | 1 | 0 | 0 | - |
| Myrmicinae | 9508 (59.6%) | 9508 | 46 | 8327 | 41 | 1181 | 5 | - |
| Ponerinae | 1720 (10.7%) | 1720 | 17 | 1196 | 16 | 524 | 1 | - |


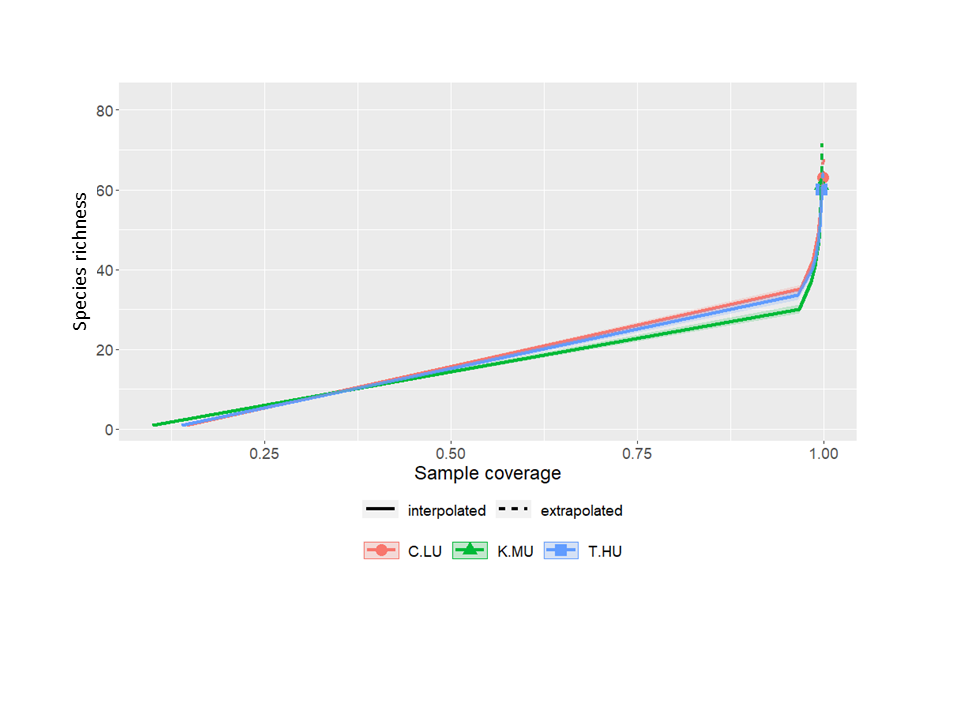


**Fig. S1. Plot shows the sample completion in three locations of different urbanization levels.** Legend: C.LU=Less-urbanized Kodagu; K.MU=Moderately-urbanized Kasaragod; T.HU=Highly-urbanized Trivandrum


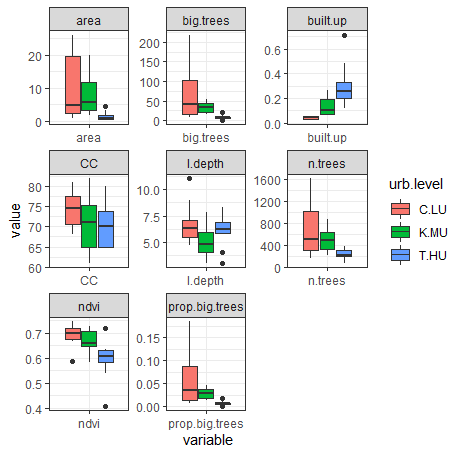


**Fig. S2. Box plots show different environmental variables of the sacred groves of three urbanization levels.** The values plotted are for the sites. Variables are area (area of the sacred grove in acres); big.trees (number of big trees in the sacred groves); CC (canopy cover on percentage scale); l.depth (leaf litter depth on cm scale); n.trees (number of total trees in the sacred groves); prop.big.trees (proportion of big trees in the sacred groves); ndvi (Normalized Difference Vegetation Matrix). Legend: C.LU: Less-Urbanized Coorg; K.MU: Moderately-Urbanized Kasaragod; T.HU: Highly-urbanized Trivandrum)


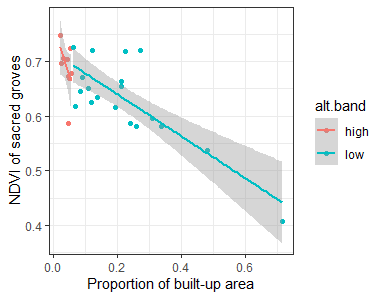


**Fig. S3. The relationship between the proportion of built-up area around the sacred groves and the quality of the sacred groves as measured by the NDVI.**


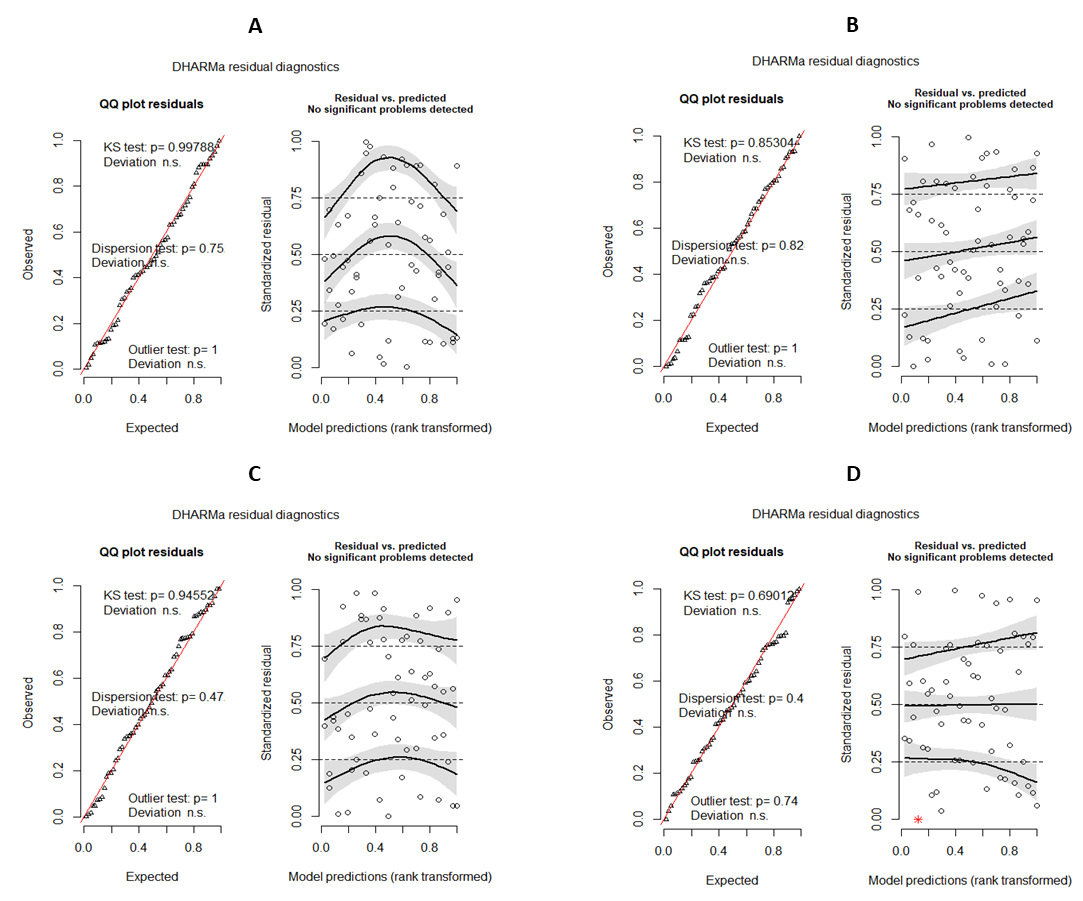


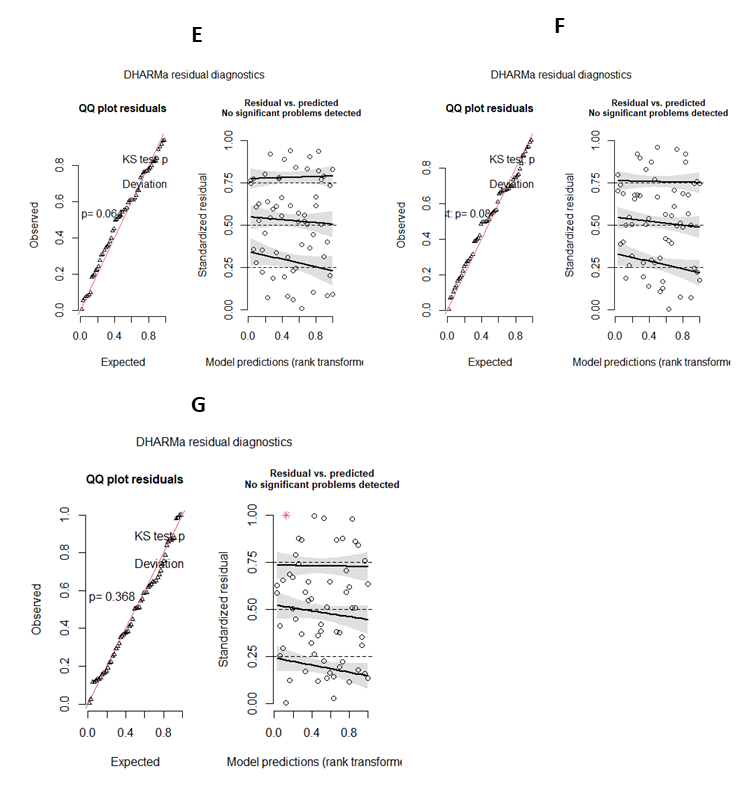

**Fig. S4. Residuals of the final fitted models produced in the R-package DHARMa. A** – *A. gracilipes* abundance, **B** – Invasive ant abundance excluding the abundance of *A.gracilipes*, **C** – Invasive ant abundance, **D** – Overall ant abundance, **E** – Overall ant rarefied richness, **F** – Native ant rarefied-richness, **G** – Native ant abundance


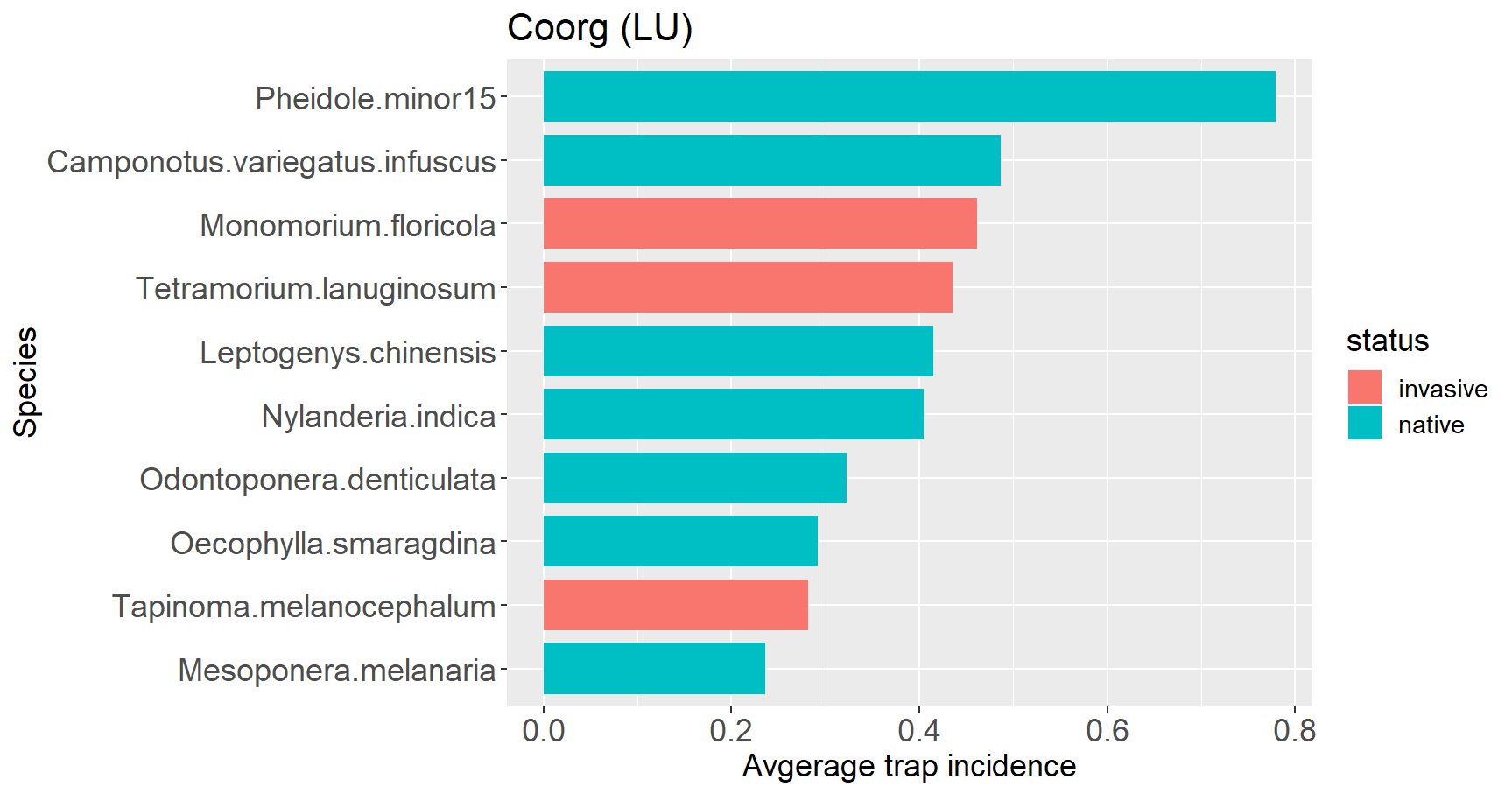


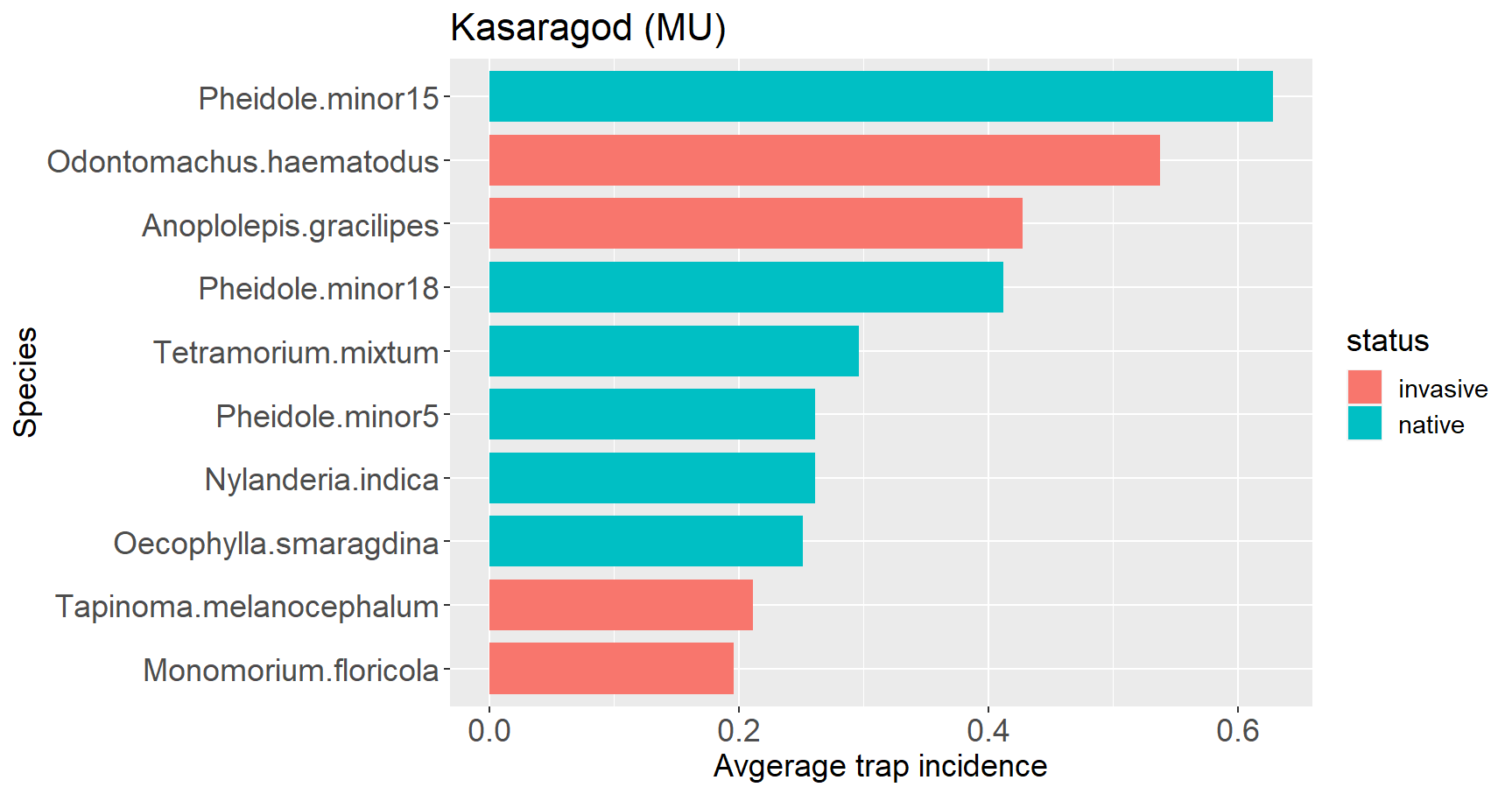


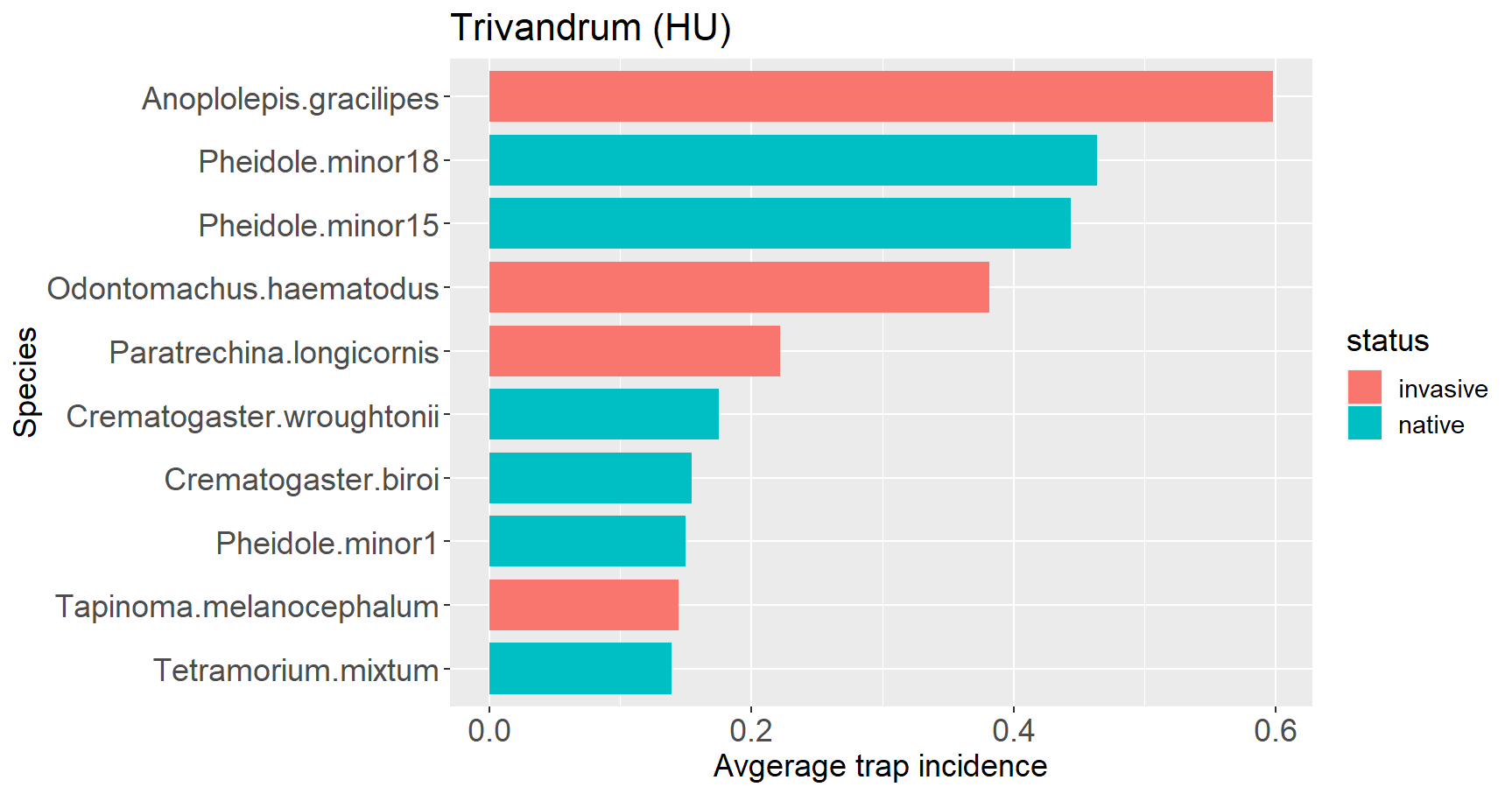


**Fig. S5**. **Site average proportional trap incidence of ten most dominating epigeic ant species of less-, moderately-, and highly-urbanized sites.**

**Fig. S6**. **Barplots show the proportional trap incidence native ants, overall invasive ants, and five dominant species of invasive ants in the sacred groves of less-urbanized (C.LU), moderately-urbanized (K.MU), and highly-urbanized locations (T.HU).**


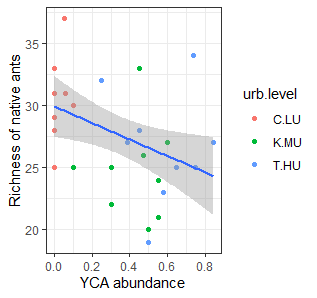

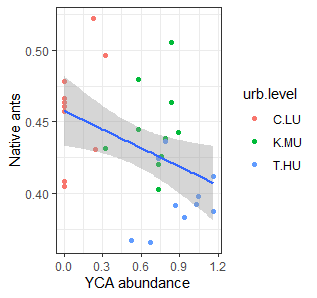

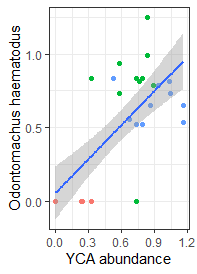

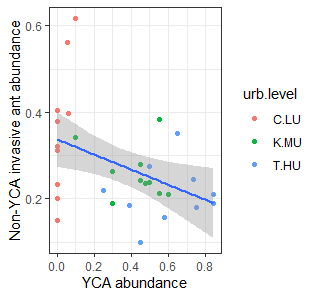


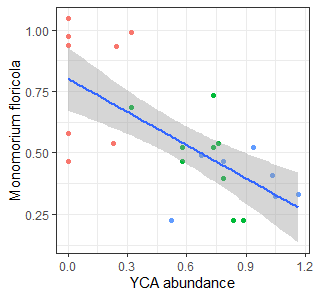

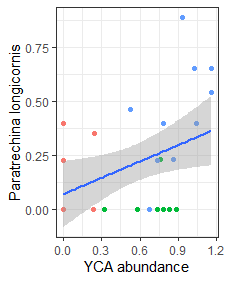

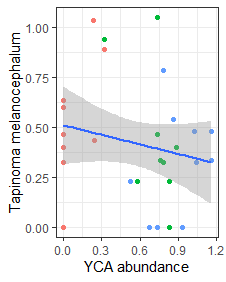

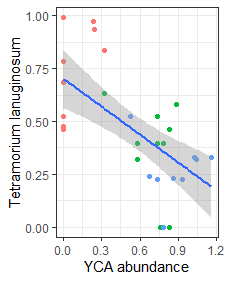


**Fig. S7. The relationships between the abundance of the Yellow-Crazy Ant and non-YCA invasive ants and native ants.** Legend: Coorg represents the less-urbanized location; Kasaragod represents the moderately-urbanized location; Trivandrum represents the highly-urbanized location. Arcsine Square-root transformed proportional trap incidence is used as the measure of abundance for both the Yellow-Crazy Ant and other ants.
